# Biallelic variants in *GTF3C3* encoding a subunit of the TFIIIC2 complex are associated with neurodevelopmental phenotypes in humans and zebrafish

**DOI:** 10.1093/braincomms/fcaf055

**Published:** 2025-02-05

**Authors:** Mohamed S Abdel-Hamid, Adeline Paimboeuf, Maha S Zaki, Fernanda Figueiredo, Sherif F Abdel-Ghafar, Sabrina Maher, Rún Friðriksdóttir, Patrick Sulem, Hákon Björn Högnason, Sigrún Hallgrímsdóttir, Catarina Falleiros N Rojas, Fernando Kok, Mohnish Suri, César Augusto P F Alves, Henry Houlden, Reza Maroofian, Shunmoogum A Patten

**Affiliations:** Medical Molecular Genetics Department, National Research Centre, Human Genetics and Genome Research Institute, Cairo 12622, Egypt; Centre Armand Frappier Santé Biotechnologie, Institut National de la Recherche Scientifique (INRS), Laval, QC, Canada H7V 1B7; Clinical Genetics Department, National Research Centre, Human Genetics and Genome Research Institute, Cairo 12622, Egypt; Mendelics Genomic Analysis, São Paulo, SP 02511-000, Brazil; Medical Molecular Genetics Department, National Research Centre, Human Genetics and Genome Research Institute, Cairo 12622, Egypt; Department of Neuroscience, Université de Montréal, Montréal, QC, Canada H3T 1J4; deCODE Genetics/Amgen Inc., Reykjavik 101, Iceland; deCODE Genetics/Amgen Inc., Reykjavik 101, Iceland; Department of Genetics and Molecular Medicine, Landspitali University Hospital, Reykjavik 101, Iceland; Department of Genetics and Molecular Medicine, Landspitali University Hospital, Reykjavik 101, Iceland; Maternal and Child Health Care Department, Faculdade de Medicina de Marília, Marília, SP 17509-054, Brazil; Mendelics Genomic Analysis, São Paulo, SP 02511-000, Brazil; Department of Clinical Genetics, Nottingham City Hospital, Nottingham NG5 1PB, UK; Division of Neuroradiology, Department of Radiology, Boston Children’s Hospital-BCH Harvard Medical School, Boston, MA 02115, USA; Department of Neuromuscular Diseases, UCL Queen Square Institute of Neurology, London WC1N 3BG, UK; Department of Neuromuscular Diseases, UCL Queen Square Institute of Neurology, London WC1N 3BG, UK; Centre Armand Frappier Santé Biotechnologie, Institut National de la Recherche Scientifique (INRS), Laval, QC, Canada H7V 1B7; Department of Neuroscience, Université de Montréal, Montréal, QC, Canada H3T 1J4

**Keywords:** *GTF3C3*, neurodevelopmental disorder, RNA polymerase III, TFIIIC, zebrafish knockout

## Abstract

RNA polymerase III transcribes essential non-coding RNAs, a process regulated by transcription factors TFIIIB and TFIIIC. Although germline variants in TFIIIC subunit genes have been described in a few patients with neurodevelopmental disorders, the associated pathogenesis and clinical spectrum are not yet well defined. Herein, we describe the identification of biallelic variants in *GTF3C3,* which encodes a key component of the TFIIIC subunit, in four patients from three unrelated families of different ethnicities collected through GeneMatcher. The patients exhibited microcephaly, developmental delay, intellectual disability and distinctive dysmorphic facies that appear recognizable in very young children. Their brain imaging showed brain atrophy with predominant cerebellar involvement, as well as hypoplasia of the frontal lobes and one patient had moderate to severe simplified gyral pattern. Seizures were observed in half of the patients. Exome/genome sequencing revealed four different *GTF3C3* variants including three missense (p.Cys172Gly, p.Val427Phe and p.Ala509Thr) and one nonsense variant (p.Arg717Ter). Missense variants were not present in known genetic databases and occurred in highly conserved residues. Knockout of the *GTF3C3* ortholog in zebrafish recapitulated the key clinical symptoms including microcephaly, brain anomalies and seizure susceptibility. We also observed reduced RNA polymerase III target gene expression in the zebrafish knockout model. This study describes a new neurodevelopmental syndrome in humans and zebrafish associated with biallelic *GTF3C3* variants and underscores the need for further research into the biological impacts of variants in TFIIIC-linked genes and their contribution to RNA polymerase III-related pathologies.

## Teaser

Abdel-Hamid *et al*. identified biallelic *GTF3C3* variants in four individuals with neurodevelopmental disorders, including developmental delay/intellectual disability, microcephaly, seizures, dysmorphic facies and brain abnormalities. Zebrafish models recapitulated key defects like microcephaly and seizures, providing insight into the disorder’s mechanisms.

## Graphical Abstract

**graphical abstract:**
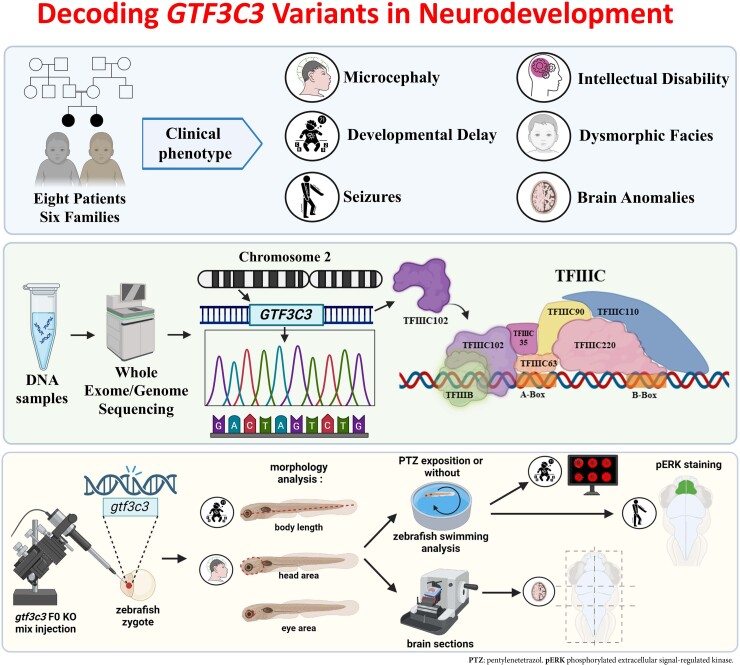

## Introduction

RNA polymerase III (POLR3), a key factor in gene expression, transcribes essential molecules such as transfer RNAs, RNA, 5S ribosomal (*RNA5S*), the spliceosomal *RNU6* (RNA, U6 small nuclear) and others.^1^ The initiation of POLR3 relies on a well-rehearsed interplay between multiple transcription factors including TFIIIB and TFIIIC. TFIIIC plays a pivotal role in the recognition and binding of gene promoters, facilitating the assembly of the pre-initiation complex. TFIIIC consists of a six-subunit complex encoded by *GTF3C1/TFIIIC220*, *GTF3C2/TFIIIC110*, *GTF3C3/TFIIIC102*, *GTF3C4/TFIIIC90*, *GTF3C5/TFIIIC63* and *GTF3C6/TFIIIC35*, respectively.^2^

There is limited knowledge on the association between germline variants in genes encoding the TFIIIC complex and human diseases. *GTF3C1* variants have been observed in a few individuals with intellectual disabilities or autism spectrum disorders.^3-5^ Similarly, biallelic variants in *GTF3C5* have been reported to be associated with various clinical features such as hypomelanosis of Ito, seizures, growth retardation, developmental delay, facial dysmorphism and abnormal brain imaging findings.^6,7^ In addition, *GTF3C3* biallelic variant have been described in four affected individuals with developmental delay, facial dysmorphism, ataxia, cerebellar atrophy and seizures.^8-10^ These studies hint at an association between TFIIIC genes and human diseases. However, conclusive evidence for specific genotype–phenotype relationships is lacking, and the biological impact of these variants necessitates further investigations.

In this report, we describe the identification of biallelic variants in *GTF3C3* in four individuals with a neurodevelopmental phenotype comprising microcephaly, developmental delay, intellectual disability, seizures and brain abnormalities on MRI. Functional analyses revealed that *gtf3c3*-knockout (KO) zebrafish display neurodevelopmental defects including microcephaly and seizures.

## Materials and methods

### Patient identification, phenotyping and genotyping

Through an international collaboration facilitated by GeneMatcher, three unrelated families of Egyptian, Icelandic and Brazilian origin were recruited and examined. Written informed consents were obtained from all three families, and the study was approved by the medical research ethics committees of each institution.

A solo exome sequencing or trio genome sequencing was performed for the probands in the respective collaborating institutions using slightly different analysis platforms according to the Burrows-Wheeler Aligner/Genome Analysis Toolkit-based pipelines. All identified *GTF3C3* variants are reported according to the transcript NM_012086.5 and classified according to the guidelines of the American College of Medical Genetics and Genomics and the Association for Molecular Pathology (ACMG–AMP). Segregation analysis and validation of the identified candidate variants were performed using Sanger sequencing.

### Zebrafish husbandry

Wild-type (WT) *Danio rerio* (AB/TL strain) and transgenic (Tg) *GFAP*:GFP [a green fluorescent protein (GFP) under the control of the astrocyte-specific glial fibrillary acidic protein (GFAP) promoter] fish were maintained at 28°C at a light/dark cycle of 12/12 h in accordance with standard practices.^11^ All zebrafish in this study were housed in groups. Fish were fed two times daily with a steady diet of Skretting^®^ Gemma Micro starting at 5 days post-fertilization (dpf). Fish larvae were fed with Gemma Micro 75, juvenile fish were fed with Gemma Micro 150 and adult fish were fed with Gemma Micro 300. Embryos were raised at 28.5°C and collected and staged as previously described.^12^ All the experiments were performed in compliance with the guidelines of the Canadian Council for Animal Care and the local Ethics Committee of INRS.

### Zebrafish study design

The primary objective of the zebrafish experiments was to assess the effects of loss of function of *gtf3c3* on zebrafish neurodevelopment. Sample size was determined based on previous experience with similar genetic, behavioural and imaging experiments. No data were excluded from the analyses. All samples used in the experiments were randomly selected. All morphological analyses, fluorescent imaging and behavioural analyses were successfully replicated and representative results were shown. Blinding during analysis was impossible as the *gtf3c3* displayed very distinct morphological phenotypes compared to WT controls. To achieve an unbiased analysis, image acquisition and analysis were randomly performed by co-authors.

### Generation of gtf3c3 F0 KO zebrafish model and overexpression/rescue experiments

Zebrafish *gtf3c3* F0 KO embryos were generated using CRISPR–Cas9^13^ following an established genome-editing protocol. Three gRNAs targeting *gtf3c3* were selected, by choosing target sequences using CHOPCHOP (https://chopchop.cbu.uib.no/) and integrated DNA technologies (https://www.idtdna.com/site/order/designtool/index/CRISPR_PREDESIGN) design tools, with higher priority given to those with fewer off-target effects. Additionally, DNA from *gtf3c3* F0 KO larvae was sequenced for the top five predicted off-target locations. Inference of CRISPR edits (ICE) analysis^14^ was used to assess cutting efficiency (Indel frequency and KO score) of gRNAs from Sanger sequencing chromatograms. Genotyping of *gtf3c3* mutant fish was performed by high-resolution melting analysis and Sanger sequencing. The sequences of primer used for genotyping and sequencing are listed in Supplementary Table 1.

For rescue and overexpression experiments of zebrafish *gftc3c* F0 KO, cDNA of WT human *GTF3C3* and homozygous missense *GTF3C3* mutations c.1279G > T (p.Val427Phe) and c.514T > G (p.Cys172Gly) were vectors purchased from VectorBuilder. Capped and polyadenylated mRNA of WT human *GTF3C* and mutants (p.Val427Phe and p.Cys172Gly) mRNA were synthesized *in vitro* using the mMESSAGE mMACHINE kit (Ambion). Embryos were either injected with 40 pg WT *GTF3C3* or mutant mRNAs at one-cell stage and compared with non-injected WT fish.

Morphological analysis was performed under a Leica S6E stereoscope. Whole-brain imaging and analysis was performed in Tg (*GFAP:*GFP) and a confocal microscope (Zeiss, LSM 780).

### Haematoxylin and eosin brain staining

Paraffin brain sections (5 µm) of 3 dpf larvae were post-fixed in 10% formol (Chaptec) for 5 min and rinsed with tap water. The sections were stained with haematoxylin (Statlab) for 4 min, washed with alcohol–hydrochloric acid and rinsed with tap water. The sections were then soaked in saturated lithium carbonate solution for 10 s and rinsed with tap water. Finally, staining was performed with Eosin Y (Statlab) for 2 min and sections mounted under coverslips with Permount mounting medium (Thermo Fisher).

### Zebrafish behavioural assays

For behavioural analysis, 5-dpf larvae were transferred individually into a 96-well plate containing 200 μL of E3 medium and habituated in the Daniovision^®^ recording chamber (Noldus) for 30 min before the start of the experiment. To assess for seizure susceptibility, 4-dpf larvae were transferred individually into a 96-well plate containing 200 μl of E3 medium or in 200 µl of 3 mM pentylenetetrazol (PTZ) and habituation was done before the treatment as described previously. Analysis was performed using the Ethovision XT 12 software (Noldus) to quantify the distance swam and swim velocity.

### p-MAPK/extracellular signal-regulated kinase staining

Larvae (4-dpf) larvae were exposed to PTZ (3 mM) treatment in the dark (for 0, 15, 30 and 45 min), rapidly fixed in 4% paraformaldehyde and kept at 4°C overnight. After fixation, larvae were rinsed several times with phosphate-buffered saline (PBS) containing 0.1% Tween 20 and then incubated in acetone (100%) for 15 min. The acetone was rinsed with PBS containing (0.3% Triton X-100), then with PBS-DT (1% bovine serum albumin, 1% dimethylsulfoxide, 1% Triton X-100) and further blocked in 5% normal goat serum in PBS-DT for 1 h. Larvae were incubated with the primary antibody anti-phospho-MAPK1/ERK2–MAPK3/ERK1 (1:500; Cell Signalling Technology, 4370S) at 4°C overnight. The samples were then rinsed several times with PBS-DT and incubated at 4°C overnight with the secondary antibody Alexa Fluor 488 goat anti-rabbit (1:1000; Invitrogen, A-11008). The larvae head were then mounted ventrally on a slide in fluoromount (ThermoFisher), imaged under a confocal microscope (Zeiss, LSM 780) and analysed using the ImageJ Fiji software.

### Quantitative real-time PCR

Total RNA was isolated from 3 dpf zebrafish larvae (20 larvae per RNA extraction) using TriReagent. One microgram of RNA was used for cDNA synthesis using cDNA vilo kit (ThermoFisher). qRT-PCR was performed with SYBR Green mix (BIORAD) with a Lightcycler96^®^ (Roche). Gene expression was analysed relative to the housekeeping gene elf1α. Primers used in quantitative PCR experiments are listed in Supplementary Table 2.

### Statistical analysis

All zebrafish experiments were done on at least three replicates (*N*) and each consisted of a sample size (*n*) of 4–52 fish. All zebrafish data values are given as means ± standard error of the mean. Significance for the zebrafish experiments was determined using either Student's *t*-test or Sidak's multiple comparisons tests. Statistical analyses were done using Prism version 9 (GraphPad Software).

## Results

### Clinical features of individuals with biallelic *GTF3C3* variants

**Figure 1:**
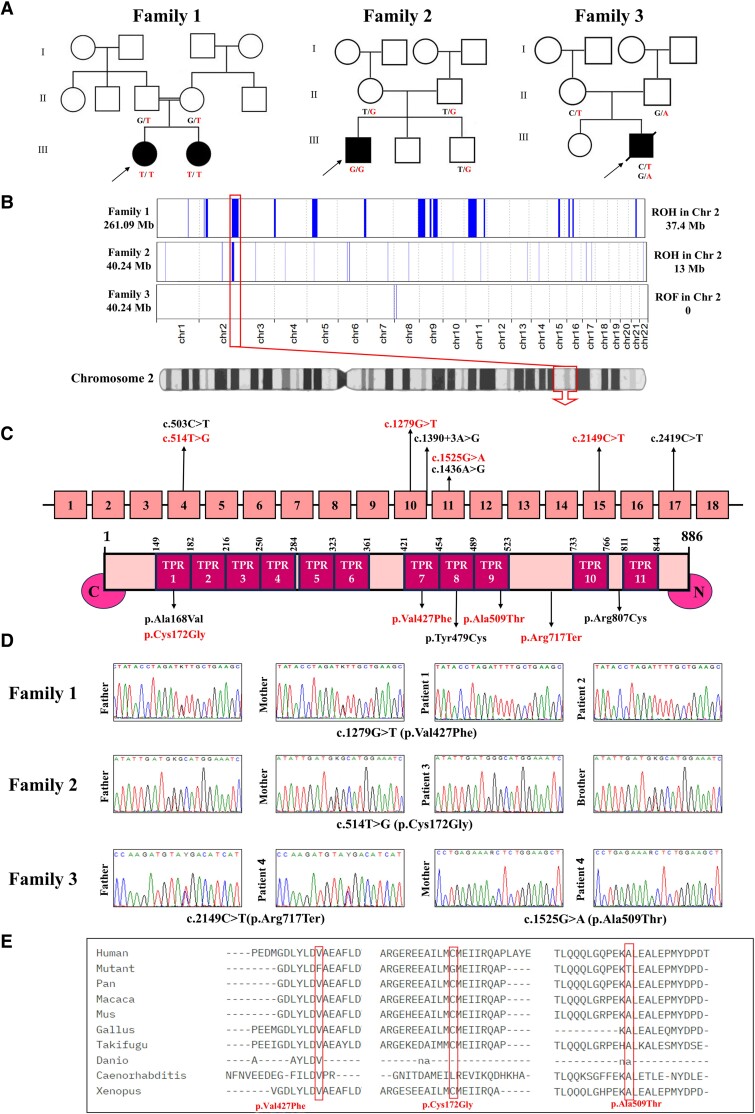
**Pedigrees and genetic findings of patients with *GTF3C3* variants.** (**A**) Pedigrees of the three families. (**B**) Homozygosity mapping using the automap tool showing the identified regions of homozygosity on chromosome 2 encompassing the *GTF3C3* gene in Families 1 and 2, in comparison to Family 3. (**C**) Schematic diagram showing the location of the identified *GTF3C3* variants in this study (in red) and those described previously (in black) and their domains. (**D**) Portions of the sequencing electropherogram showing the segregation of the *GTF3C3* variants in the three families. (**E**) The conservation of the three new missense variants p.Cys172Gly, p.Val427Phe and p.Ala509Thr.

**Figure 2:**
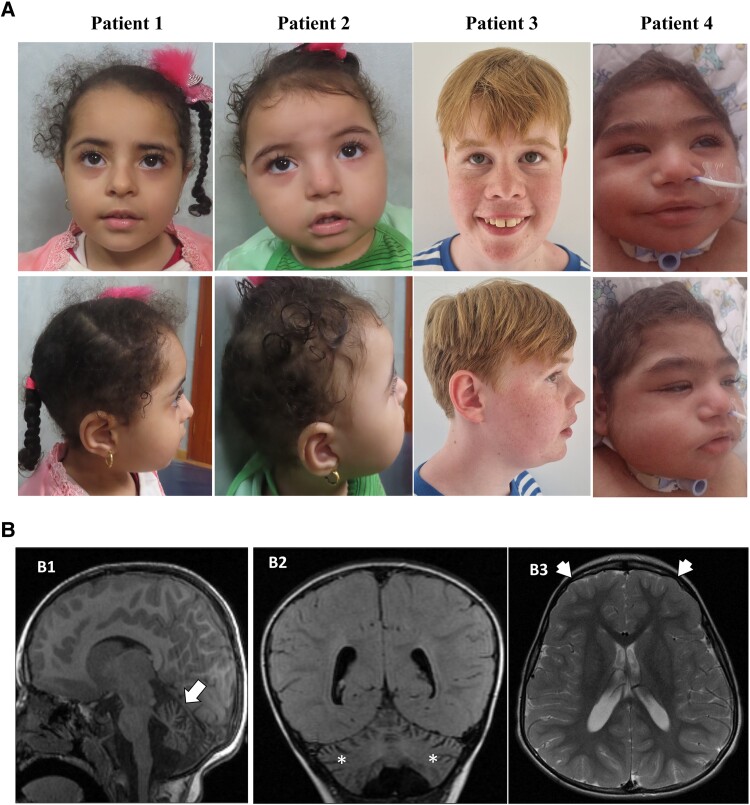
**Facial features and brain imaging of patients.** (**A**) Facial photographs of four patients showing distinctive facial features: Patient 1—bifrontal narrowing, long eyelashes, upslanting palpebral fissures, hypertelorism, full cheeks, full nasal tip, tented upper lip, full lower lip; Patient 2—round face, frontal prominence, bifrontal narrowing, long eyelashes, hypertelorism, short nose, broad nasal bridge, full nasal tip, full cheeks, tented upper lip, full lower lip; Patient 3—long face, bifrontal narrowing, infraorbital crease, smooth philtrum, full lower lip; Patient 4—microcephaly, low anterior hairline, bifrontal narrowing, hypertrichosis over forehead, synophrys, long eyelashes, hypertelorism, upslanting palpebral fissures, epicanthus, full cheeks, short nose, full nasal tip, tented upper lip, full lower lip. (**B**) Brain MRI images: B1 and B2—sagittal T1-weighted image and coronal fluid-attenuated inversion recovery images demonstrating selective atrophy of the cerebellar cortex, involving both the cerebellar vermis (arrow, B1) and hemispheres (asterisk, B2); B3—axial T2WI showing relatively small frontal horns and hypoplastic frontal lobes (short arrows, B3) compared with the rest of the brain.

The study investigated a neurodevelopmental disorder affecting four patients from three unrelated families. The detailed clinical and genetic data are summarized in Table 1. Pedigrees show the presence of consanguinity in 2/3 families (Fig. 1A). The major clinical findings observed in the patients were microcephaly (4/4), developmental delay (4/4), intellectual disability (4/4) and dysmorphic facies (4/4; Fig. 2A**)**. The facial photographs of our patients were compared with the available photos of previously published patients and there may be a recognizable face in very young children with this disorder which is best seen in Patient 2 and the patient described by Anazi *et al*.^8^ The most frequently observed dysmorphic features include narrow forehead/bifrontal narrowing (100%), hypertelorism (67%), long eyelashes (56%), full/broad nasal tip (89%), tented upper lip (56%) and thick lower lip vermilion/full lower lip (78%). The detailed dysmorphic facial features of the four patients are depicted in Supplementary Table 3. Seizures were observed in two patients (2/4). One patient experienced generalized tonic–clonic seizures associated with fever while the other had tonic seizures that were controlled with antiepileptic drugs. Two patients exhibited skeletal abnormalities including short stature, genu valgus, pes valgus and sacral fovea. Other rare findings were nystagmus (1 patient), reduced vision (1 patient) and hearing loss (1 patient). On MRI scans, three out of the four patients exhibited varying degrees of brain volume loss. Among these, two patients showed predominant involvement of the cerebellar cortex, including the vermis and hemispheres and one patient had also associated hypoplasia of the frontal lobes (Fig. 2B). One patient presented with malformative features characterized by severe simplified gyral pattern of the brain.

**Table 1:** The demographic, clinical and genetic data of patients with biallelic *GTF3C3* variants

Family/individual		F1–P1	F1–P2	F2–P3	F3–P4	Reuter *et al.*^9^		Anazi *et al.*^8^	Papuc *et al.*^10^
**Gender/age at last examination**		Female/2 years 8 months	Female/1 year 9 months	Male/17 years	Male/2 years (died at 2 years and 4 months)	Female/13 years	Female//22 years	Female/5 years 9 months	Female/24 years
***GTF3C3* variant (NM_012086.5)**		c.1279G > T (p.Val427Phe)	c.1279G > T (p.Val427Phe)	c.514T > G (p.Cys172Gly)	c.2149C > T (p.Arg717Ter)/ c.1525G > A (p.Ala509Thr)	c.1436A > G (p.Tyr479Cys)	c.1436A > G (p.Tyr479Cys)	c.1390 + 3A > G	c.503C > T (p.Ala168Val)/c.2419C > T (p.Arg807Cys)
**Zygosity**		Homozygous	Homozygous	Homozygous	Compound heterozygous	Homozygous	Homozygous	Homozygous	Compound heterozygous
**Origin/consanguinity**		Egypt/+	Egypt/+	Iceland/−	Brazil/−	Turkey/+	Turkey/+	Saudi Arabia/+	Switzerland/Europe/−
**Growth at birth**	**Weight**	3.2 kg (−0.4SD)	3 kg (−0.8SD)	3.2 kg (−1.1SD)	2.1 kg (−2.3SD)	NA	NA	NA	NA
	**Length**	47.5 cm (−0.8SD)	48 cm (−0.6SD)	51 cm (0.1SD)	41 cm (−3.4SD)	NA	NA	NA	NA
	**OFC**	34 cm (−0.5SD)	33.5 cm (−0.8SD)	33 cm (−2.2SD)	29 cm (−3SD)	NA	NA	NA	NA
**Growth at last examination**	**Weight**	13 kg (−0.4SD)	13 kg (+1SD)	45 kg (−3.2SD)	7.5 kg (−4.2SD)	NA	NA	NA	NA
	**Length**	89 cm (−1.1SD)	85 cm (+0.1SD)	158.7 cm (−3.2SD)	NA	NA	NA	NA	NA
	**OFC**	44 cm (−2.8SD)	41 cm (−4.3SD)	49.8 cm (−3.5SD)	34 cm (−10.3SD)	NA	NA	NA	NA
**Microcephaly**		+	+	+	+	NA	NA	+	+
**Dysmorphic facies**		+	+	+	+	+	+	+	+
**Developmental delay**		+ Global delay	+ Global delay	+	+	+	+	+	+
**Intellectual disability**		+ Severe ID	+ Severe ID	+ Moderate ID	+	+ Moderate ID	+ Mild ID	+ Severe ID	+ Severe ID
**Seizures/type/onset**		+/GTC/ at 1year	−	−	+/Tonic seizures/12 months	+/Febrile seizures / NA	+/Absence seizure/NA	−	+/Myoclonic, GTC/NA
**EEG findings**		Normal at 2 years	Normal at 1 year	NA	Disorganization of electrical activity	Abnormal in infancy/early childhood	Still abnormal at 22 years	Abnormal EEG	Diffuse slow and monomorphic rhythm, epileptiform discharges
**Neurological characteristics**		Hypotonia, unsteadiness,	Hypotonia, unsteadiness, drooling	Mild hypotonia	Hypertonia	Ataxic gait, Dystonic movement of hands	NA	Hypotonia, head ataxia	Limb spasticity, choreoathetotic movement
**Neuroimaging features**		Mild diffuse brain volume loss with slightly thin CC and predominant CBA. Ex-vacuo DLV more on left side.	Mildly DLV, thin CC, mild CBA	Diffuse simplified gyral pattern with pontocerebellar hypoplasia	Slightly short CC, with relative hypoplasia of the frontal lobes, and diffuse CBA	NA	NA	Prominent cortical sulci, Dandy Walker variant, aplasia/hypoplasia of the CC	Hypoplastic CC, severe cerebral and marked cerebellar atrophy with simplified gyral pattern
**Gastrointestinal/feeding problems**		−	−	+Chronic constipation	+Nasoenteral tube	+	NA	+	+
**Other findings**		Autistic features	Autistic features	Bilateral syndactyly of toes II and III, genu valgus, pes valgus, retinopathy	Nystagmus, hearing loss, thrombocytopenia, anaemia	Hearing loss	Hearing Loss	−	Scoliosis

CBA, cerebellar atrophy; CC, corpus callosum; DLV, dilated lateral ventricles; EEG, electroencephalography; F, family; GTC, generalized tonic-clonic; ID, intellectual disability; NA, not available; OFC, occipitofrontal circumference; P, patient.

### Identification of biallelic variants of *GTF3C3*

Exome and genome analysis identified four different variants in the *GTF3C3* including three missense and a stop gain variant. In Family 1, a homozygous missense variant c.1279G > T (p.Val427Phe) in exon 10 was identified. Another homozygous missense variant in exon 4 (c.514T > G, p.Cys172Gly) was detected in Family 2. Interestingly, homozygosity mapping of Families 1 and 2 showed that the identified *GTF3C3* variants reside in homozygous regions of 37.40 and 13 Mb, respectively (Fig. 1B). Family 3 exhibited compound heterozygous variants, a maternally inherited missense variant (c.1525G > A, p.Ala509Thr) and a paternally inherited stop gain variant (c.2149C > T, p.Arg717Ter) in exons 11 and 15, respectively (Fig. 1C and D). The three missense variants were absent in gnomAD database v.4 in either the heterozygous or homozygous state, indicating their rarity in the general population. However, the c.2149C > T (p.Arg717Ter) was present in 64 heterozygous individuals with an allele frequency of 0.00003971. Importantly, all missense variants affected highly conserved amino acids (Fig. 1E) and were predicted to be ‘deleterious’ by multiple in silico tools **(**Supplementary Table 4). A structural model of GTF3C3 was generated using Alphafold and PremPS predicted significant changes in the 3D structure of the protein due to the three substitutions of p.Cys172, p.Val427 and p.Ala509 (Supplementary Fig. 1).

### Neurodevelopmental defects including seizure susceptibility in gtf3c3 KO zebrafish

To further gain insights about the pathogenetic mechanism of biallelic *GTF3C3* variants, we examined the effect of knocking out *gtf3c3 in vivo* in the zebrafish model. The zebrafish genome encodes a single *gtf3c3* ortholog that shares 65.75% nucleotide identity with human *GTF3C3*. We generated a *gtf3c3* biallelic zygotic KO model using CRISPR–Cas9.^13^ We used the F0 KO method^13^ to induce biallelic mutations in zebrafish *gtf3c3* gene. Three sets of Cas9–gRNA ribonucleoproteins to target *gtf3c3* (Supplementary Fig. 2A) was used to generate biallelic zygotic KO directly in the injected embryos (*gtf3c3* F0 KO). Sanger sequencing followed by ICE analysis of injected embryos revealed mutations at the targeted loci, with a high percentage of indels leading to premature stop codons (Supplementary Fig. 2B–D). Real-time quantitative PCR analysis, revealed a significant decrease in *gtf3c3* mRNA expression in *gtf3c3* F0 KO fish (Supplementary Fig. 2E and F).

**Figure 3:**
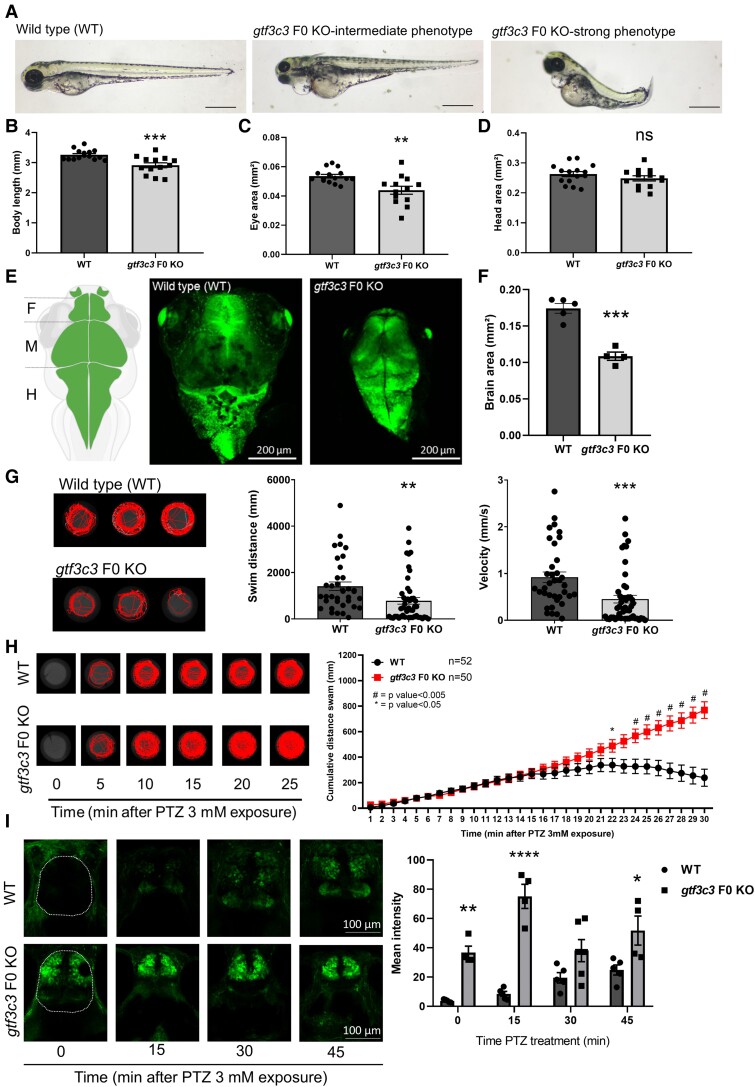
**Modelling *gtf3c3* KO in zebrafish recapitulates patients’ clinical features.** (**A**) Morphology of zebrafish WT, and *gtf3c3* F0 KO larvae at 3 dpf. Scale bars: 500 µm. Body length (**B**), head size (**C**) and eye size (**D**) of WT and *gtf3c3* F0 KO larvae at 3 dpf (*n* = 13–15). (**E**) Schematic representation and image of transgenic (Tg) *GFAP*:GFP head in dorsal view of 3-dpf WT and *gtf3c3* F0 KO larvae. Scale bar: 200 μm. (**F**) Quantification of brain size of *gtf3c3* F0 KO larvae (3 dpf, *n* = 4) compared to WT (3 dpf, *n* = 5) showed a significant reduction that could reflect microcephaly. (**G**) Representative swimming tracks, and quantification of swimming and velocity of WT (*n* = 36) control and *gtf3c3* F0 KO (*n* = 48) fish at 5 dpf. (**H**) Representative swim traces of control WT fish and *gtf3c3* F0 KO followed through time with PTZ treatment at 4 dpf. Quantification of swimming distance with PTZ treatment normalized to fish without treatment showed that *gtf3c3* F0 KO (*n* = 50) larvae exhibited seizure-like behaviour after 15 min of PTZ treatment compared to WT (*n* = 52). (**I**) Neuronal activity induced by PTZ treatment (3 mM; 0, 15, 30 and 45 min) in 4-dpf larvae analysed by imaging p-MAPK/ERK staining. Quantification of mean intensity fluorescence of p-MAPK/ERK staining in telencephalic region (surrounded in red) showed a significant increase in *gtf3c3* F0 KO larvae compared to WT at 0-, 15- and 45-min treatment of PTZ. According to the neuronal activity, we can suggest that *gtf3c3* F0 KO (0, 15, 30 and 45 min, *n* = 4–6) larvae exhibit seizure-like behaviour relative to the WT (*n* = 5). All data are represented as the mean ± SEM. Statistical significance was calculated by Student's *t*-test or Mann–Whitney test, or Sidak' multiple comparisons tests. *P < 0.05, **P < 0.01, #*P* < 0.005, ***P < 0.001, ****P < 0.0001; ns, not significant. *n* represents number of fish.

At 3 dpf, *gtf3c3* F0 KO larvae displayed a smaller body size (intermediate phenotype), with some of the larvae exhibiting shorter body length with slightly curved body axis (strong phenotype; Fig. 3A and B) as compared with WT controls. Additionally, the *gtf3c3* F0 KO 3-dpf fish displayed a smaller eye size (Fig. 3C) compared with WT controls. At the gross morphology level, no major changes were observed in the head size (Fig. 3D). Sequencing of DNA from *gtf3c3* F0 KO larvae for the top five predicted off-target locations showed no alterations to the base sequence of these potential off-target sites (Supplemental Fig. 2G). These data confirm that our F0 KO approach generates biallelic mutations specifically in *gtf3c3* and they influence the expression of the morphological phenotypes.

We further examined the brain structure in 3 dpf whole-mount larvae and at the histological level on transverse brain sections at 3 dpf using hemotoxylin and eosin/H&E staining. We found significant structural differences and smaller brain size in 3-dpf brains from *gtf3c3* F0 KO relative to WT larvae (Fig. 3E and F; Supplementary Fig. 3A–D), consistent with microcephaly and brain malformations observed in the cohort.

Locomotor activity in *gtf3c3* F0 KO was assessed by automated swimming tracking. Zebrafish *gtf3c3* F0 KO larvae at 5 dpf exhibited reduced basal locomotion compared with WT controls (Fig. 3G). Administration of the pro-convulsive gamma-aminobutyric acid receptor antagonist PTZ (3 mM) to *gtf3c3* F0 KO larvae provoked a marked increase in locomotion compared with controls. In particular, the relative activity of *gtf3c3* F0 KO larvae in PTZ, normalized to basal activity, increased to a much greater extent than that observed in control larvae (Fig. 3H). To further assess whether *gtf3c3* F0 KO larvae have distinct sensitivities to PTZ-induced neuronal hyperactivation, we examined the activity of MAPK/extracellular signal-regulated kinase (ERK) signalling by quantifying phosphorylated MAPK1/ERK2-MAPK3/ERK1 (p-MAPK/ERK) following PTZ treatment. The *gtf3c3* F0 KO larvae showed a strikingly elevated p-MAPK/ERK staining in the telencephalic brain region (Fig. 3I). Noteworthy, even in the absence of PTZ, p-MAPK/ERK activity was significantly higher in *gtf3c3* F0 KO fish compared with WT controls (Fig. 3I), suggesting an overactive basal brain activity in the mutant fish. Altogether, these findings suggest an abnormal neural excitatory/inhibitory balance in *gtf3c3* F0 KO fish, consistent with an increased seizure susceptibility.

**Figure 4:**
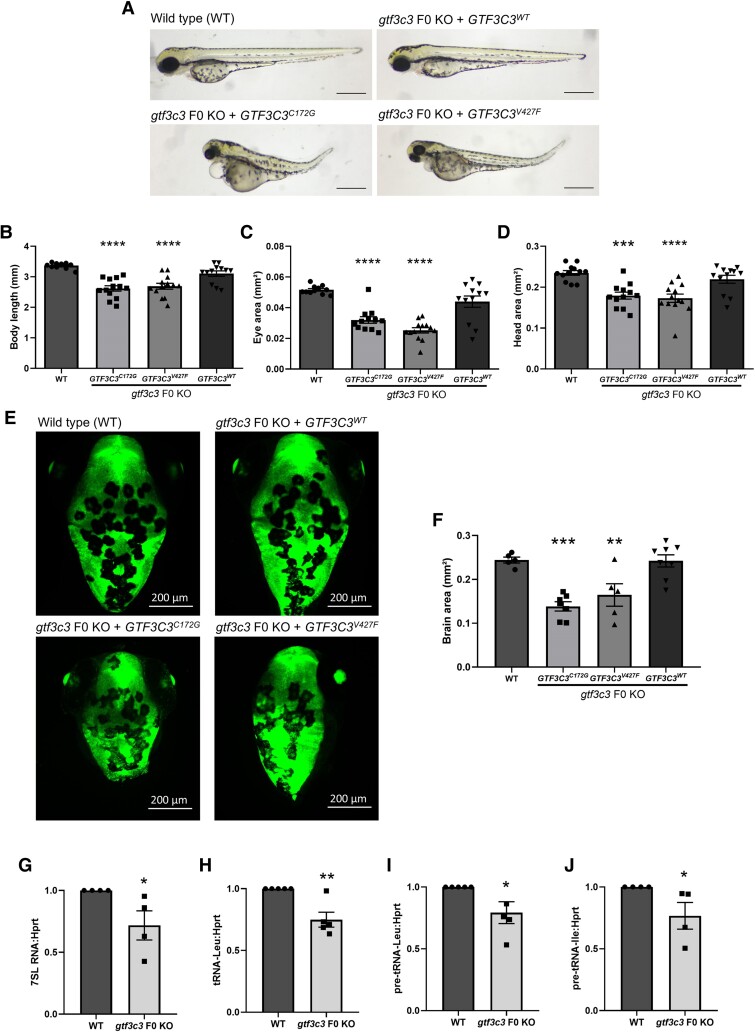
**Effect of *GTF3C3* mutations on POLR3 target gene expression.** (**A**) Morphology of zebrafish WT, *gtf3c3* F0 KO + *GTF3C3^WT^*, *gtf3c3* F0 KO+*GTF3C3^C172G^* and *gtf3c3* F0 KO + *GTF3C3^V427F^* larvae at 3 dpf. Scale bars: 500 µm. Body length (**B**), eye size (**C**) and head size (**D**) of WT, *gtf3c3* F0 KO + *GTF3C3^WT^*, *gtf3c3* F0 KO + *GTF3C3^C172G^* and *gtf3c3* F0 KO + *GTF3C3^V427F^* at 3 dpf (*n* = 12–13). (**E**) Dorsal view of GFAP 3 dpf larvae WT, *gtf3c3* F0 KO + *GTF3C3^WT^*, *gtf3c3* F0 KO + *GTF3C3^C172G^* and *gtf3c3* F0 KO + *GTF3C3^V427F^*. Scale bars: 200 µm. (**F**) Head area of GFAP 3 dpf larvae WT, *gtf3c3* F0 KO + *GTF3C3^WT^*, *gtf3c3* F0 KO + *GTF3C3^C172G^* and *gtf3c3* F0 KO + *GTF3C3^V427F^*(*n* = 12–13). (**G–J**) Quantitative real-time PCR. (**G**) 7SL RNA, (**H**) *tRNAleu*, (**I**) *pre-tRNAleu* and (**J**) *pre-tRNAile* expression relative to the expression of the Pol II target gene *hrpt*, in pooled 3 dpf (*n* = 20 for each pool) *gtf3c3* F0 KO compared to pooled WT larvae (*N* = 4–5). All data are represented as the mean ± SEM. Statistical significance was calculated by Mann–Whitney test, or Dunnett's multiple comparisons tests. *P < 0.05, **P < 0.01; #*P* < 0.005, ***P < 0.001; ****P < 0.0001; ns, not significant. *n* represents number of fish, *N* represents number of experimental repeats.

To confirm if the observed phenotypes are caused by the loss of function of *gtf3c3*, we employed a rescue approach using human *GTF3C3* mRNA (*GTF3C3^WT^*). Injection of human *GTF3C3^WT^* rescued of *gtf3c3* F0 KO morphological phenotypes such as the eye, head and body size, and microcephaly (Fig. 4A–F). However, missense variants p.Val427Phe (*GTF3C3^V327F^*) and p.Cys172Gly (*GTF3C3^C172G^*) in *GTF3C3* were unable to efficiently rescue the morphological phenotypes in F0 knockouts (Fig. 4A–F). Altogether, these data confirm that gtf3c3 has conserved functions in zebrafish and human and that the observed phenotypes are caused by loss of *gtf3c3* function.

### Altered Pol III function in gtf3c3-KO zebrafish

To determine whether the *gtf3c3* biallelic mutations disrupted POLR3 function, we quantified the levels of POLR3 target gene RNAs in WT and *gtf3c3* F0 KO larvae.^15,16^ We observed a significant reduction in the levels of 7SL (Fig. 4G) and tRNA-leu (Fig. 4H) in 3 dpf *gtf3c3* F0 KO larvae relative to the levels of the POLR2 (Pol II) target gene *hprt*. Noteworthy, no change was observed in the level of *hprt* expression in *gtf3c3* F0 KO larvae. To further assess POLR3 function, we examined its transcription rate by examining the levels of pre-tRNAs in WT and *gtf3c3* F0 KO larvae.^16,17^ Using quantitative PCR, we measured pre-tRNA levels in WT and *gtf3c3* F0 KO larvae, relative to the POLR2 transcribed gene *hprt* mRNA. Interestingly, we found a marked reduction in levels of both of the pre-tRNAs examined (pre-tRNA-leu and pre-tRNA-ile; Fig. 4I and J) in *gtf3c3* F0 KO larvae compared with controls. Altogether, these findings suggest that POLR3 transcription is reduced in *gtf3c3* F0 KO fish.

## Discussion

In this study, we identified biallelic variants in *GTF3C3* in four individuals with a neurodevelopmental disorder characterized by microcephaly, global developmental delay, intellectual disability, facial dysmorphism, seizures and brain anomalies including most commonly cerebellar atrophy, and less frequently diffuse simplification of the gyral pattern of the brain. *GTF3C3/TFIIIC102* encodes an essential component of the TFIIIC transcription factor complex involved in the regulation of RNA polymerase III activity.

POLR3 is considered the largest of the three eukaryotic RNA polymerases. It consists of 17 subunits and the active site of the enzyme is formed by POLR3A/RPC1 and POLR3B/RPC2, which are parts of the 10-subunit core. This core includes two subunits shared with POLR1 (POLR1C and POLR1D) and five subunits shared with POLR1 and POLR2 (POLR2E, POLR2F, POLR2H, POLR2K and POLR2L), along with POLR3K/RPC11. The remaining seven subunits of PORL3 form different sub-complexes. The stalk complex, consisting of POLR3H and CGRP-receptor component protein, is involved in POLR3 initiation, whereas a heterotrimer composed of POLR3C, POLR3F and POLR3G, as well as a heterodimer formed by POLR3D and POLR3E, are important for both initiation and termination of transcription.^18,19^

The significance of POLR3 in human health was underscored by the discovery of the association between pathogenic variants in its subunits or associated transcription factors and a broad range of genetic disorders. Biallelic variants in *POLR3A*, *POLR3B*, *POLR1C* and *POLR3K* cause POLR3-related hypomyelinating leukodystrophy (HLD), which is characterized by hypomyelination, cerebellar atrophy, motor dysfunction, dental and endocrine abnormalities and ocular anomalies.^20-23^ In addition to POLR3-HLD, biallelic variants in *POLR1C* and *POLR1D* lead to Treacher Collins syndrome, which presents mainly with craniofacial abnormalities.^24^ Wiedemann–Rautenstrauch syndrome is another severe condition linked to variants in *POLR3A*, *POLR3B* and *POLR3GL* and is characterized by intrauterine growth restriction, facial dysmorphia, lipodystrophy and dental anomalies.^25,26^ Furthermore, biallelic variants in *POLR3GL* cause a syndrome with short stature, motor delay, oligodontia, dysmorphic facies and growth retardation.^27^

Similarly, pathogenic variants in genes encoding the essential transcription factors for POLR3 have been linked to a variety of human diseases. Variants in *BRF1*, a subunit of TFIIIB, result in cerebellofaciodental syndrome, which is characterized by intellectual disability, speech impairment, craniofacial abnormalities, dental anomalies, short stature and cerebellar hypoplasia.^28^  *De novo* repeat expansions in *TBP*, another TFIIIB subunit, cause spinocerebellar ataxia type 17.^29^ Additionally, variants in *BDP1*, the third subunit of TFIIIB, have been associated with hereditary hearing loss.^30^

Variants in the TFIIIC subunits genes have been reported in a limited number of patients with neurodevelopmental disorders. A few studies have reported the associations between *GTF3C1* and *GTF3C2* variants with autism spectrum disorders and intellectual disability.^3-5,31^ Additionally, a biallelic variant in the *GTF3C5* was first described in a patient presenting with developmental delay, dysmorphic facies, hypomelanosis of Ito, seizures, growth retardation and brain abnormalities.^6^ Recently, *GTF3C5* variants have been described in four patients from three families with a complex neurodevelopmental phenotype including intellectual disability, developmental delay, growth retardation, facial dysmorphism, delayed bone age, skeletal anomalies, dental anomalies and predominant cerebellar atrophy, similar to the brain imaging features observed in some of the patients described in this study.^7^

So far, only four patients from three unrelated families harbouring recessive *GTF3C3* variants have been sporadically described.^8-10^ Many clinical characteristics are shared between those patients and the four new patients described in this report such as facial dysmorphism, developmental delay, intellectual disability, brain atrophy with predominant cerebellar involvement, hypoplasia of the frontal lobes and diffuse simplification of the gyral pattern of the brain. Based on these observations, it appears that variants in the TFIIIC subunit genes, specifically *GTF3C3* and *GTF3C5*, share many overlapping clinical features (Supplementary Table 5). Additionally, variants in *BRF1*, which encodes a part of the TFIIIB complex, show some clinical similarities. This finding suggests a potential common pathway or mechanism underlying these disorders, given that TFIIIC and TFIIIB are interacting together for the proper initiation of transcription by POLR3.^2^

Loss of function of *gtf3c3* in zebrafish resulted in neurodevelopmental defects further supporting a role for Gtf3c3 in the development and function of the central nervous system. Importantly, these results are consistent with a role of GTF3C3 in brain function, motor function and susceptibility to seizures. Human GTF3c3 and its zebrafish orthologous protein share conserved tetrarico peptide repeat domains, tetraricopeptide-like helical domain superfamily and the transcription initiation factor IIIC TFIIIC, polypeptide 3-related region. Our findings of reduced levels of the POLR3 target genes in *gtf3c3* KO are consistent to those observed in *slj* zebrafish mutant bearing a deletion mutation in the POLR3 subunit, *polr3b.*^16^ Thus, the *gtf3c3* KO zebrafish model can serve as an excellent system to further understand the brain defects and their underlying mechanisms. The relative abundance of *gtf3c3* mRNA in F0 KO zebrafish was significantly decreased, suggesting a loss of mutant transcript via nonsense-mediated decay. Nevertheless, F1 germline *gtf3c3* KO mutant should be established for future experiments including to investigate alterations in molecular signatures in the zebrafish *gtf3c3* KO brain.

## Conclusion

In conclusion, this study further confirms the association of biallelic variants in *GTF3C3* with an autosomal recessive neurodevelopmental disorder featuring intellectual disability, microcephaly, brain anomalies and in some cases seizures. *In vivo* studies of loss of function of *gtf3c3* in zebrafish show in addition to microcephaly, an exacerbated motor response to PTZ and higher p-MAPK/ERK brain activity, features that are consistent with increased seizure susceptibility. TFIIIC is a basal transcription factor complex for POLR3 and plays a key role in chromatin organization. Biallelic variants in *GTF3C3* likely have an impact on the functioning of the TFIIIC complex and affect the precise control of the expression of key neurodevelopmental genes.

## Supplementary Material

fcaf055_Supplementary_Data

## Data Availability

All supporting data in this study are available from the corresponding author on request, subject to appropriate privacy and ethical restrictions.
